# Contextualizing results of randomized trials in smoldering myeloma

**DOI:** 10.1093/oncolo/oyaf216

**Published:** 2025-07-17

**Authors:** Ghulam Rehman Mohyuddin, Aaron M Goodman, Rajshekhar Chakraborty

**Affiliations:** Division of Hematology and Hematological Malignancies, Huntsman Cancer Institute, University of Utah, Salt Lake City, UT 84106, United States; Division of Blood and Marrow Transplantation, University of California San Diego, CA 92037, United States; Division of Hematology, Columbia University Irving Cancer Center, New York City, NY 10027, United States

**Keywords:** smoldering myeloma, early intervention, daratumumab, myeloma, cancer prevention

Smoldering myeloma (SMM) is a precursor stage of multiple myeloma (MM) and is present in one in 200 individuals over the age of 40 years.^1^ Historically, the diagnosis of MM has required the presence of end-organ damage (such as high **C**alcium levels, **R**enal failure, **A**nemia, or lytic **B**one lesions, CRAB criteria); however, changes to diagnostic criteria in 2014 led to reclassification of some patients previously classified as high-risk SMM as MM.^2^ These patients were estimated to have an 80% risk of progression to MM at 2 years from diagnosis and thus reclassified as MM based on this risk.^2^ In view of this, the external validity of trials that enrolled patients with SMM prior to 2014 remains uncertain. Nevertheless, we now have several randomized trials that have enrolled patients after these diagnostic changes (Table 1). In this editorial, we aim to contextualize the results of those randomized controlled trials for SMM and highlight our current approach to management.

**Table 1.** Characteristics of key randomized trials in SMM.

RCT/Author	Treatment		Inclusion criteria	Baseline imaging	Surveillance strategy in control arm	% of SLiM included	Primary endpoint results	Overall survival	Post-protocol therapy received at MM diagnosis in control arm
	Experimental arm	Control arm							
QUIREDEX/Mateos *et al*	Lenalidomide-Dexamethasone (Rd)	Observation	(a) BMPC≥10% and one of the following-IgG≥3 g/dL IgA≥2 g/dL, or BJP>1g/24 hours ***or*** (b) One of the above and ≥95% phenotypically aberrant PCs in BMPC compartment with immunoparesis	Skeletal survey	Serum and urine monoclonal protein every 4 weeks (*no surveillance imaging*)	Not provided; however, 100% of accrual prior to 2014 diagnostic reclassification	Median TTP: 2.1 vs 9.5 years in observation vs Rd, respectively (HR 0.28, 95% CI: 0.18-0.44, *P* <.0001)	Median OS: 8.5 years vs not reached in observation vs Rd, respectively (HR 0.57, 95% CI: 0.34-0.95, *P* = .032)	(a) Bortezomib-based combinations without lenalidomide (48%) (b) Lenalidomide-based therapy (28%)
ECOG	Lenalidomide	Observation	BMPCs ≥10% and abnormal FLC ratio (<0.26 or >1.65)	MRI spine/pelvis	None	(a) 8.2% with FLC ratio>100 (with iFLC >10 mg dL^−1^) (b) 3.3% with BMPCs ≥60%	HR for PFS with lenalidomide vs observation (only CRAB events included in PFS): 0.28; 95% CI, 0.12 to 0.62; *P* = .002	No OS benefit at a median follow-up of ∼3 years	Not provided
AQUILA	Daratumumab	Observation	BMPCs ≥10% and one of the following: M-spike ≥3 g dL^−1^ IgA isotype Immunoparesis FLC ratio 8 to less than 100 %BMPC >50% but <60%	MRI spine/pelvis, Whole-body CT (preferred), PET/CT	MRI spine/pelvis, Whole body CT (preferred), PET/CT	None	5-year PFS with Daratumumab vs Active Monitoring: 63.1% vs 40.8% (HR 0.49; 95% CI, 0.36-0.67; *P* <.001)	5-year OS with Daratumumab vs Active Monitoring: 93% vs 86.9% (HR 0.52; 95% CI, 0.27-0.98)	First line MM therapy among 105 patients in control arm that required therapy: (a) VRd/KRd: 32 (30.5%) (b) Total patients with anti-CD38-containing regimens, including all subsequent treatments: 35 (17.9%)

Abbreviations: BMPC: bone marrow plasma cells; BJP: Bence–Jones protein; TTP: time to progression; HR: hazard ratio; FLC: free light chain; CRAB: hypercalcemia, renal insufficiency, anemia, and bone lesions.

Two trials had previously shown that intervention with lenalidomide (with or without dexamethasone) can decrease the likelihood of progression for patients with high-risk SMM to MM.^3^^,^^4^ The first trial by the Spanish Group began enrolling in 2007, well before the use of advanced imaging and diagnostic reclassification of MM.^4^ As such, a substantial proportion of participants in that trial would have MM today. Although an overall survival (OS) advantage was seen in this trial upon extended follow-up, most patients in the control arm did not receive lenalidomide (or other contemporary therapies) when they experienced disease progression, and this trial was not statistically powered for OS either.^5^

The E3A06 trial comparing lenalidomide to observation also began enrolling prior to the reclassification of SMM to MM, although the diagnostic reclassification occurred during the trial period.^3^ This trial demonstrated that there was a decreased risk of progression with lenalidomide compared to observation. However, it was unclear whether early treatment led to prevention of asymptomatic bone lesions picked up on imaging, or whether actual fractures or irreversible organ damage were prevented. Furthermore, this trial was not powered to show an OS difference, and at 3 years of follow-up, approximately 70% of patients in the observation arm were without evidence of disease progression. Additionally, a patient in the safety run of the study developed myelodysplastic syndrome/acute myeloid leukemia upon follow-up.

Although both these trials did demonstrate a decreased risk of progression for early intervention, whether these interventions improved survival or quality of life remained unanswered, setting the stage for the newest randomized trial in this space.

The most recent randomized trial in this space is the AQUILA trial evaluating daratumumab for SMM.^6^  Table 1 summarizes characteristics of AQUILA and the two previously discussed trials. This trial randomized 390 patients with high-risk SMM to either daratumumab or observation and demonstrated a benefit of the primary endpoint of progression-free survival (PFS), with a PFS at 5 years of 63.1% vs 40.8%.

This trial is noteworthy for being conducted with advanced imaging, as opposed to skeletal surveys, and enrolling patients fully after the recent diagnostic reclassifications in 2014.^2^ The active surveillance strategy and mode of progression in AQUILA trial, along with three large contemporary real-world studies have been summarized in Table 2.

**Table 2.** Characteristics of progression in the AQUILA trial and key retrospective datasets.

Variables (*n*; %, unless otherwise stated)	AQUILA trial (*n* = 360)		Heidelberg study (*n* = 447)		Mayo Clinic Study (*n* = 406)		Greek Study (*n* = 427)	
	Daratumumab Arm (*n* = 194)	Active Surveillance Arm (*n* = 196)						
Baseline imaging during screening/diagnosis	Lytic lesion assessment by LDWBCT (preferred) or PET/CT or CT; Focal lesion assessment by MRI spine and pelvis		WB DW-MRI		Not specified		LDWBCT, WB DW-MRI, or PET/CT	
Monitoring for MDEs	MRI spine/pelvis every 6-12 months^a^ for the first 3 years, then every 12 months, and at biochemical/suspected PD; LDWBCT or PET/CT for lytic lesion assessment performed only if triggered by new focal lesion on MRI spine/pelvis, biochemical PD, or suspected PD to myeloma		WB DW-MRI yearly^b^; LDWBCT for lytic lesion assessment performed in case of evolving M-protein/sFLC ratio, or suspected progression (*85% underwent ≥1 WB DW-MRI*)		Frequency and preferred modality of follow-up imaging not specified		LDWBCT, WB DW-MRI, or PET/CT yearly; clinical and laboratory evaluation every three months for the first year, every four months for the second year, with follow-up beyond two years being every four months in high-risk SMM and every 6 months in low-intermediate risk SMM	
Median follow-up (years)	5.4		5.8		3.4-5.0		3.0	
**Progression details**			**Entire Cohort (*n* = 447)**	**HR-SMM (*n* = 82; 18%)**	**Non-HR SMM (*n* = 226)^c^**	**HR-SMM (*n* = 90; 22%)^a^**	**Entire Cohort (*n* = 427)**	**HR-SMM (*n* = 73; 17%)**
Percent progressed to SLiM-CRAB (%)	34.5	50.5	28.9	56.1	39.0	71.8	10	3-year risk of progression: 43%
Renal insufficiency	0 (0)	0 (0)	9 (2.0)	2 (2.4)	5 (2.6); *4 (2.1%)* with irreversible renal failure	3 (4.2); *1 (1.4%)* with irreversible renal failure	1 (0.2)	0 (0)
Anemia	2 (1.0)	14 (7.1)	28 (6.3)	11 (13.4)	21 (11.0)	18 (25.4)	11 (2.6)	7 (9.6)
Bone disease	10 (5.2)	18 (9.2)	60 (13.4)	14 (17.1)	30 (15.7)	19 (26.8)	11 (2.6)	8 (10.96)
Fractures	Not reported	Not reported	Not reported	Not reported	4 (6.8)	5 (7.0)	1 (0.2)	Not reported
≥60% clonal BMPCs	5 (2.6)	16 (8.2)	40 (8.9)	9 (11.0)	8 (4.2)	12 (16.9%)	6 (1.4)	Not reported
Serum FLC ratio ≥100	33 (17.0)	33 (16.8)	47 (10.5)	21 (25.6)			12 (2.8)	Not reported
>1 focal lesion on MRI	12 (6.2)	16 (8.2)	32 (7.2)	11 (13.4)	None	None	6 (1.4)	Not reported
SLiM-only progressions (% of total progression events)	Not reported	Not reported	40	Not reported	14	24	43	37.5

a 71/90 patients were observed, hence the denominator is 71 for all MDEs.

b A recent version of protocol (September 2024) states the frequency of MRIs once a year, in contrast to an uploaded version of protocol from 2017 on the NEJM website.

c 191/226 were observed, hence the denominator is 191 for all MDEs.

Abbreviations: LDWBCT: low-dose whole-body CT; HR-SMM: high-risk smoldering multiple myeloma; WB DW-MRI: whole-body diffusion-weighted magnetic resonance imaging; PD: progressive disease.

In our opinion, five key caveats exist regarding this study. First, it appears that treatment with daratumumab prevented asymptomatic laboratory and imaging findings rather than true morbidity. Since the SLiM criteria have been adopted in 2014,^2^ more contemporary data indicates that the true risk of progression to myeloma with end organ damage for patients with SLiM criteria is ∼30% in the next 2 years rather than 80%.^7^ Additionally, data from real-world studies suggest that 14%-40% of all progression events in contemporary SMM are SLiM-only progressions.^8–10^ Unlike previous studies of lenalidomide, in the AQUILA trial, a clear description of the nature of progression events was provided. It appears that progression events were significantly driven by asymptomatic SLIM events. This is exemplified best by 5 occurrences of ≥60% plasma cells in the daratumumab arm, vs 16 in the control, an asymptomatic finding that was discovered only on frequent bone marrow sampling.

Anemia progression events were more common in the control arm compared to the daratumumab arm (14 [7%] vs 2 [1%]). Amongst the patients who developed anemia, the median change in hemoglobin was 1.7 g dL^–1^, and no corresponding adverse event of fatigue was reported.^11^ Given that this drop in hemoglobin was mild and did not appear to be symptomatic, it remains unclear whether treatment with daratumumab to prevent this drop in hemoglobin would be worth the risk and cost of therapy. A total of 28 (16.9%) patients (amongst the 166 patients who experienced a PFS event) across both arms experienced bone events as their progression criteria to myeloma, of which only three patients had symptomatic bone pain, and two of these patients were in the daratumumab arm.^11^ This highlights how even amongst the patients who experienced CRAB events in this trial, morbidity was rare. Three recent retrospective datasets of patients with SMM on observation highlight the low incidence of morbidity (Table 2),^9^^,^^10^^,^^12^ and prior data also suggest that progression events in patients with SMM are almost always not morbid.^13^^,^^14^ Approximately 20% of patients who were diagnosed with myeloma in this trial did not initiate treatment, raising questions about the clinical meaningfulness of progression as currently defined in myeloma, using the SLIM criteria in SMM trials.^11^

Second, this trial already appears to be demonstrating improved overall survival with 5-year overall survival at 93.0% with daratumumab (vs 86.9% with active surveillance). For a trial testing a drug in SMM that is already known to be efficacious in myeloma, the control arm should receive effective therapy incorporating daratumumab upon progression.^15^ This trial enrolled from December 2017 through May 2019, but patients in the control arm would have likely progressed in the few years that followed, during a time when daratumumab had already shown efficacy for newly diagnosed myeloma. The ALCYONE trial had led to daratumumab approval for newly diagnosed myeloma by the Food and Drug Administration (FDA) as early as May 2018.^16^ We believe it was a missed opportunity by the sponsor of the trial not to have provided daratumumab-based regimens (or contemporary triplet therapy) to the control arm at progression to newly diagnosed myeloma. Of a total of 105 patients in the control arm who went on to receive therapy, only 29 (27.8%) received bortezomib/lenalidomide/dexamethasone, and only 18% of patients received a regimen containing anti-CD38 therapy. It is crucial we know what treatments were given to the individuals in the control arm who died from progressive myeloma, as well as how many people received daratumumab at any point in their disease course. It must also be noted that the trial was not powered for OS, and there appears to be an imbalance in the number of deaths due to reasons other than myeloma (12 in daratumumab arm, 17 in observation arm).

Third, in this trial, the best available imaging modality to detect bone disease in myeloma- whole-body diffusion-weighted MRI (WB DW-MRI) imaging—was not utilized. While not universally utilized yet as a standard of care, several retrospective and prospective studies have now demonstrated that WB DW-MRI has a significantly greater sensitivity than positron emission tomography (PET)/CT or WB CT in detection of focal myeloma lesions.^17–19^ Furthermore, the protocol mandated only spine and pelvis MRI imaging at baseline, rather than whole-body MRI, which can potentially miss focal lesions in long bones, ribs, and skull. Up to half of lesions can be missed by doing only a spine MRI, as opposed to a whole-body MRI.^20^ Another prospective study has demonstrated that the discordance between WB DW-MRI and PET/CT is greatest in long bones, with a 45% greater likelihood of picking up focal lesions in long bones with the former.^17^ Furthermore, patients were recommended to get a low-dose whole-body CT yearly, with the use of a PET scan optional.

Fourth, although it is tempting to consider this trial a comparison of receiving relatively nonintensive and finite duration daratumumab now vs receiving intensive therapy for myeloma (incorporating multiple drugs for prolonged periods of time), we believe this analogy is incorrect. 46.4% of patients in the control arm had still not needed any myeloma therapy at approximately 5 years. It also appears that the use of daratumumab did not permanently avert future myeloma therapy, as 33.6% of patients in the daratumumab arm still needed further myeloma therapy, and no plateau has been observed in the PFS curve of the daratumumab arm.

A final key limitation is that the definition of high-risk utilized in the study did not align with currently used risk stratification systems, as only 40.5% of all patients enrolled in the trial were considered high-risk by the Mayo 2018 and International Myeloma Working Group risk stratification systems.

How would we counsel patients given the data we have for both lenalidomide and daratumumab for SMM today? In settings where there is access to diffusion-weighted whole-body MRI, and where better therapy can be given when people develop myeloma, it is reasonable to expect that outcomes for patients will be much better than the control arm of these trials. Daratumumab does reduce the risk of asymptomatic lab and imaging changes from developing; however, the risk of morbid progressions appears to be extremely low with this modern strategy of surveillance imaging. Should patients elect to receive therapy for SMM, we do believe daratumumab is a safer choice than lenalidomide due to its safety profile and lack of secondary malignancy risk.

In summary, despite various successful randomized controlled trials of interventions for smoldering myeloma, we believe morbidity to be very low with modern surveillance strategies, and with modern therapy available upon progression, it is unknown if early intervention improves OS for smoldering myeloma. Our preferred option remains to offer patients close observation in lieu of treatment.
